# Validation study of the Functional Assessment of Cancer Therapy-Cognitive Function-Version 3 for the Portuguese population

**DOI:** 10.1186/s40359-022-01018-w

**Published:** 2022-12-14

**Authors:** Ana F. Oliveira, Isabel M. Santos, Sofia Fernandes, Pedro Bem-Haja, Ana Torres

**Affiliations:** 1 Center for Health Technology and Services Research of the Health Research Network (CINTESIS@RISE), Department of Education and Psychology, University of Aveiro, 3810-193 Aveiro, Portugal; 2 William James Center for Research (WJCR), Department of Education and Psychology, University of Aveiro, 3810-193 Aveiro, Portugal; 3 Department of Education and Psychology, University of Aveiro, 3810-193 Aveiro, Portugal; 4 Department of Psychology and Education, Faculty of Human and Social Sciences, University of Beira Interior, 6200-209 Covilhã, Portugal

**Keywords:** FACT-Cog-v3, Perceived cognitive functioning, Cancer patients, Validation study

## Abstract

**Background:**

Cancer-related cognitive impairment is a common and potentially debilitating symptom experienced by patients with non-central nervous system (CNS) cancers, with negative impact on their quality of life. The Functional Assessment of Cancer Therapy-Cognitive Function-Version 3 (FACT-Cog-v3) is the most extensively used instrument specifically developed to evaluate cognitive complaints in adult cancer patients. Nevertheless, this self-report measure is not yet validated for the Portuguese population. Therefore, the purpose of this study was to evaluate the psychometric properties of the FACT-Cog-v3 among patients with non-CNS cancers in Portugal.

**Methods:**

The validation study was conducted based on a convenience sample of 281 patients with non-CNS cancers, aged between 18 and 65 years, recruited online. A confirmatory factor analysis (CFA) was used to test the factor structure of the Portuguese FACT-Cog-v3 version; internal consistency analysis was also conducted. The European Organization for Research and Treatment of Cancer Quality of Life Questionnaire Core-30 (EORTC QLQ-C30–version 3) and the Hospital Anxiety and Depression Scale (HADS) were also used to test the concurrent, convergent, and discriminant validity of the scale.

**Results:**

CFA supported a four-factor model with good fix indexes and internal consistencies: perceived cognitive impairments (α = 0.97), comments from others (α = 0.92), perceived cognitive abilities (α = 0.93), and impact on quality of life (α = 0.92). Concurrent, convergent, and discriminant validities were confirmed. Moderate and strong correlations were found between the FACT-Cog-v3 subscales and the QLQ-C30 cognitive functioning subscale. Good convergent validity, with moderate correlations, was found between the FACT-Cog-v3 subscales and the HADS-A, HADS-D, and QLQ-C30 fatigue, sleep disturbance, and global health status subscales. Acceptable discriminant validity, with weak and moderate correlations, was demonstrated between the FACT-Cog-v3 subscales and the QLQ-C30 pain and nausea/vomiting subscales.

**Conclusions:**

The Portuguese FACT-Cog-v3 version can be considered a reliable and valid measure to assess cognitive concerns of patients with non-CNS cancers, with relevance for research and clinical practice.

**Supplementary Information:**

The online version contains supplementary material available at 10.1186/s40359-022-01018-w.

## Background

Cancer-related cognitive impairment (CRCI) refers to cognitive problems related with cancer and cancer treatments, and is commonly experienced by patients throughout the disease trajectory [1, 2]. Although subtle, problems with short-term and working memory, attention, processing speed, and executive functions can have a significant impact on various domains of the quality of life (QoL) of patients, including work and social life [3]. Given the consequences of CRCI and its high prevalence (from 22 to 41% in patients with non-central nervous system (CNS) cancers compared to healthy patients; [4]), the identification of individuals with CRCI is necessary to guarantee adequate supportive care to those who need it [1].

Cognitive function can be assessed by formal neuropsychological tests (*objective cognitive function*) and subjective assessments (*perceived/subjective/self-reported cognitive function,* indifferently named in this study as *perceived cognitive functioning* or PCF) [5–7]. Both objective and PCF are important outcomes in research and clinical practice. Traditionally, formal neuropsychological tests have been viewed as the “gold standard” measure of cognitive function [5], detecting subtle impairments in non-clinical populations [8]. However, these tests can be burdensome for patients and researchers and may not be sensitive to detect subtle changes in cancer patients [8, 9]. Subjective assessment, through the administration of self-report questionnaires, can be a more practical approach, being effective and valid to measure patients’ PCF [7, 9, 10]. Although subjective assessment shows a limited correlation with neuropsychological evaluation (see [5, 6] for several factors that may contribute to this difference), it is clinically very useful to understand patient distress, perception of cognitive functioning, and to identify patients with subtle deficits who may benefit from a neuropsychological assessment and/or close monitoring [10]. Thus, some authors advocate that subjective assessment is even more relevant than neuropsychological tests [11]. Furthermore, previous systematic reviews verified a moderate to strong association between self-reported cognitive symptoms and patient reported-outcomes, such as anxiety, depression, fatigue, and lower health status [2, 5].

Most PCF questionnaires were not developed for and have not been properly validated with cancer patients [5, 7]. Two measures have been most commonly used in the literature to assess PCF in cancer patients, namely the cognitive functioning subscale of the European Organization for Research and Treatment of Cancer Quality of Life Questionnaire Core-30 (EORTC QLQ-C30-version 3, briefly QLQ-C30) [12] and the Functional Assessment of Cancer Therapy-Cognitive Function-Version 3 (FACT-Cog-v3) [7]. The QLQ-C30 is a measure of QoL and comprises only two items assessing cognitive function, namely memory and concentration. Therefore, considering that this questionnaire does not assess other cognitive domains and does not provide additional information on the impact of the cognitive changes on QoL, its use as a unique indicator of PCF may result in an underestimation of the extent of cognitive difficulties [5]. Consequently, a more comprehensive and multi-dimensional measure is potentially more valid [5, 10]. In this context, the FACT-Cog-v3 [7] is one of the most well-known and the most commonly used instruments [13], both in research and in clinical settings, specifically developed to evaluate cognitive complaints in adult cancer patients [1, 5].

The FACT-Cog-v3 is a relatively brief measure and seems to be one of the most promising self-report instruments to evaluate these specific concerns, incorporating multiple dimensions such as perceived cognitive impairments (CogPCI), comments from others (CogOth), perceived cognitive abilities (CogPCA), and impact on quality of life (CogQoL). This scale was originally developed in English [7] and has been widely administered across clinical settings and validated across different cultures and languages, including French [14], Chinese [15], Korean [16], Japanese [17], Turkish [18], and English [9, 19]. These validation studies have shown good psychometric qualities, including reliability, validity, and demonstration of cross-cultural adequacy.

To our knowledge, in Portugal, there are no validated comprehensive scales to assess PCF among cancer patients. The Functional Assessment of Chronic Illness Therapy (FACIT) team, who developed the FACT-Cog-v3, has also developed other health outcomes measures specific for cancer patients. Among those is the Functional Assessment of Cancer Therapy-General (FACT-G), which has been adapted and validated to the Portuguese population [20]. However, this instrument only assesses quality of life in cancer patients and does not provide any measure of PCF. Therefore, the aim of this study was to evaluate the psychometric properties of the FACT-Cog-v3 among patients with non-CNS cancers in Portugal. The factor structure and internal consistency of this version were explored. Furthermore, the relationship between the FACT-Cog-v3 and theoretically related constructs was examined to determine the concurrent, convergent, and discriminant validity of the measure.

## Methods

### Participants

A convenience sample of 281 patients with non-CNS cancers completed the FACT-Cog-v3 online. The inclusion criteria were: (1) age between 18 and 65 years old; (2) diagnosis of non-CNS cancer; (3) undergoing or having received treatments for cancer; (4) ability to read and write Portuguese; and (5) Portuguese nationality. Patients with (1) psychiatric or communication disorders, and/or other serious medical condition; (2) CNS metastasis; and (3) diagnosis of dementia, epilepsy, brain injury (stroke, head injury), and drug or alcohol abuse, were excluded since these conditions might impact on cognitive functioning. Of the total sample, 266 participants additionally filled out the QLQ-C30 and, of those, 258 participants also filled out the Hospital Anxiety and Depression Scale (HADS) (see Measures section for a description of these instruments).

### Procedure

Volunteer cancer patients were recruited through online advertisement disseminated across Portugal. An online survey (LimeSurvey®) located on a server from the University of Aveiro was used to collect data from participants. Participants were a self-selected sample who replied to advertisements posted on social media (Facebook), specifically in support groups, blogs/forums, cancer-related information groups, and pages of national cancer associations that accepted to collaborate in the dissemination of the study, targeting Portuguese adult cancer patients; national cancer associations were also invited to collaborate in disseminating information about the study by e-mail to their associates. Advertisements invited potential participants to access a link to the survey. Those who clicked on the link were then given detailed information about the study’s goals, inclusion criteria, and ethical statements. Participants were informed that their participation was voluntary and confidentiality of the data was ensured. Cancer patients who agreed to the study conditions provided their informed consent by clicking on the “Yes” option to the question “Do you accept to participate in this study?”. The survey was open for four months, between January and April 2021. The protocol took approximately 30 min to complete. Participants’ ethical treatment was safeguarded, in accordance with the Declaration of Helsinki [21] and the guidelines of the American Psychological Association [22]. The Ethics and Deontology Committee of the University of Aveiro (22 January 2020/ No. 30/2019) approved all the procedures of this study.

### Measures

Participants completed a global self-report questionnaire assessing sociodemographic (e.g., age, education, occupation) and clinical variables (e.g., cancer diagnosis, treatments, brain injuries).

**Fig. 1:**
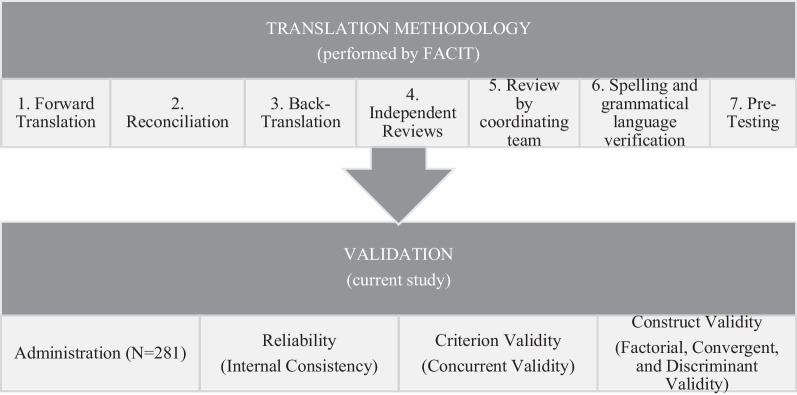
Overview of the process of translation and validation of the Portuguese version of the Functional Assessment of Cancer Therapy-Cognitive Function-Version 3 (FACT-Cog-v3)

The version 3 of the FACT-Cog [7] used in this study was translated into universal Portuguese by the FACIT team, using an iterative methodology [23, 24]. For the present study, authorization was requested from FACIT to test its psychometric properties. Figure 1 presents an overview of the translation process performed by FACIT as well as a schematic representation of the validation process described in the present article.

The FACT-Cog-v3 is a 37-item self-response measure to assess cognitive concerns of cancer patients, consisting of four subscales. For CogPCI (20 items; 0–80) and CogOth (4 items; 0–16) items, the patient indicates how often the situation occurred during the last 7 days, on a 5-point Likert scale (*“0* = *Never”* to *“4* = *Several times a day”*); and for CogPCA (9 items; 0–36) and CogQoL (4 items; 0–16), a 5-point Likert scale (*“0* = *Not at all”* to *“4* = *Very much”*) is used to indicate the severity of each situation taking into account the last week. Although two items of CogPCI and two items of CogPCA are not currently scored under the FACT-Cog-v3 scoring algorithm, according to FACIT, they may be included if some additional analyses (i.e., internal consistency and individual item-total correlation coefficients) are conducted to confirm that the items have a good fit with the scale. Therefore, the 37 items were used in this study to test its psychometric properties [19]. Except for the CogPCA subscale, negatively worded items are reverse scored prior to summing all the items. Higher scores indicate better PCF and better QoL. The reliability and validity of these scores have been established [14, 16], including the preliminary evaluation of the Portuguese version that revealed good psychometric properties regarding reliability and concurrent and convergent validity [25].

The QLQ-C30 [12, 26] is a 30-item self-response questionnaire that was used to assess health-related QoL. This scale includes a global health status/QoL subscale, functional and symptom subscales, and single items. Each of the items is scored on a 4-point Likert scale (“*1* = *Not at all*” to “*4* = *Very much*”), except the items of the global health/QoL subscale (modified 7-point linear analogue scale). The scores for each subscale range from 0 to 100, with higher scores for functional scales and global health/QoL representing better functioning and QoL, while higher scores in the symptom subscales and single items are indicative of worse symptoms. Of interest in this study was the cognitive functioning, global health/QoL, fatigue, and sleep disturbance subscales. Good psychometric properties were found on the Portuguese validation study [26]. In this study, the subscales used have shown acceptable Cronbach’s alpha: Cognitive Functioning = 0.79, Fatigue = 0.88, Pain = 0.88, Nausea/Vomiting = 0.70, and Global Health Status/QoL = 0.91.

This study included use of the HADS [27, 28], a 14-item self-response questionnaire, useful in recognizing emotional components of physical illness. The HADS consists of two subscales, each with seven items, one measuring anxiety (HADS-A) and one measuring depression (HADS-D); these items are answered on a 4-point Likert scale. Each subscale has a score ranging 0–21 points; higher scores indicate a higher level of anxious and depressive symptoms. Good psychometric properties were found on the Portuguese validation study [28]. In this study, Cronbach’s alpha was acceptable (0.86) for both subscales.

### Statistical analysis

Statistical analyses were performed with the Statistical Package for the Social Sciences (IBM SPSS, version 28.0; IBM SPSS, Inc., Chicago, IL) and with the lavaan package for R [29, 30].

Descriptive statistics were first calculated for sample’s demographic and clinical characteristics. Measurement characteristics, i.e., mean scores, standard deviations (SD), and range, are presented for each subscale.

Reliability, through internal consistency, was measured using the following techniques and cut-off recommendations: mean of the inter-item correlation (adequate if > 0.30), corrected item-total correlation (adequate if > 0.50) [31], and Cronbach’s alpha (acceptable if > 0.70 and high if > 0.90) [32–34].

To test criterion validity of the scale, concurrent validity was established via correlation coefficients between the scores of the FACT-Cog-v3 and the QLQ-C30 cognitive functioning subscale.

Construct validity was determined by factorial, convergent, and discriminant validity. Confirmatory factor analysis (CFA) was used to test the hypothesis that the construct of PCF, as assessed by the FACT-Cog-v3, is composed of four separate factors of CogPCI, CogOth, CogPCA, and CogQoL [7]. Mardia’s Test was performed to assess the multivariate normality of the sample. Regarding sample size requirements for CFA, rules-of-thumb vary from five to 10 subjects per variable, including a minimum of 100 subjects [34] or a range of 200–300 individuals [35, 36]. A CFA using weighted least squares with mean and variance adjustment (WLSMV) estimator was conducted. We considered the following goodness-of-fit indices and respective cut-off recommendations for good adjustment [31, 37–40]: Chi-Square (*χ*2); Comparative Fit Index (CFI; 0.90 ≤ CFI ≤ 0.95); Tucker-Lewis Index (TLI; 0.90 ≤ TLI ≤ 0.95); Root Mean Square Error of Approximation (RMSEA; 0.05 ≤ RMSEA ≤ 0.08); and Standardized Root Mean Square Residual (SRMR ≤ 0.08). Local model fit was assessed through the items’ standardized factor loadings (λ ≥ 0.50) and individual reliability (R^2^ ≥ 0.25) [31, 40].

Convergent and discriminant validity were assessed using the Fornell and Larcker [41] criterion and by correlations with external criteria. Convergent validity of the measurement model can be assessed by the average variance extracted (AVE; AVE ≥ 0.50) and construct reliability (CR) for each factor (CR ≥ 0.70) [41], and discriminant validity is supported when the AVE for a construct is greater than the squared interconstruct correlations [31]. Convergent validity was also assessed by examining the correlations between FACT-Cog-v3 subscales and HADS and QLQ-C30 fatigue, sleep disturbance, and global health status subscales. Discriminant validity was further examined through the correlation between FACT-Cog-v3 subscales and QLQ-C30 pain and nausea/vomiting subscales.

Following the guidelines presented by Ratner [42], the correlations were classified as weak (0–0.3), moderate (0.3–0.7), and strong (> 0.7–1.0).

All significance tests were conducted using a significance level of *p* < 0.05.

## Results

### Participants

The demographic and clinical characteristics of the sample are displayed in Table 1. Cancer patients were 18–65 years-old and the mean age was 45.97 years. The most frequently reported cancer diagnosis was breast cancer (62.7%), followed by Hodgkin lymphoma and colorectal cancer (both 6.0%). More than half of the cancers (68.3%) were diagnosed during the last 5 years. More than 80% of the sample had undergone surgery (82.9%) and chemotherapy (80.8%). Presently, 57.3% of the sample has completed the treatments, while 29.9% are still receiving hormone therapy.

**Table 1:** Sociodemographic and clinical characteristics of the sample (N = 281)

Characteristic	N (%)
Age (Years) [Mean (SD), range]	45.97 (9.00), 18–65
Gender	
Female	268 (95.4%)
Male	13 (4.6%)
Marital status	
Single	55 (19.6%)
Married	148 (52.7%)
Cohabitation	34 (12.1%)
Divorced or separated	43 (15.3%)
Widowed	1 (0.4%)
Children (Yes)	211 (75.1%)
Education	
Less than 4 years of education	1 (0.4%)
1st Cycle (4th year complete)	5 (1.8%)
2nd Cycle (6th year complete)	4 (1.4%)
3rd Cycle (9th year complete)	13 (4.6%)
Secondary Education (12th year complete)	87 (30.9%)
Higher Education—Bachelor’s degree	117 (41.7%)
Higher Education—Master’s degree	48 (17.1%)
Higher Education—Doctoral degree	6 (2.1%)
Education (Years) [Mean (SD), range]	15.56 (3.87), 4–29
Occupation	
Working (part- and full-time)	185 (66.1%)
Medical leave	56 (20.1%)
Unemployed	20 (7.2%)
Student	6 (2.2%)
Retired	14 (5.0%)
Monthly income (euros)	
< 500€	28 (10%)
500€–999€	104 (37.0%)
1000€–1499€	77 (27.4%)
1500€–1999€	45 (16.0%)
> 2000€	27 (9.6%)
Cancer type	
Bladder	1 (0.4%)
Lung	8 (2.8%)
Uterus	3 (1.1%)
Leukemia	5 (1.7%)
Hodgkin Lymphoma	17 (6.0%)
Non-Hodgkin Lymphoma	10 (3.6%)
Melanoma	3 (1.1%)
Multiple Myeloma	3 (1.1%)
Cervical	6 (2.1%)
Colorectal	17 (6.0%)
Stomach	2 (0.7%)
Breast	176 (62.7%)
Ovarian	11 (3.9%)
Skin non-melanoma	3 (1.1%)
Thyroid	6 (2.3%)
Other (e.g., nasopharynx, liver, sarcoma)	10 (3.9%)
Year of cancer diagnosis	
≤ 2000	3 (1.1%)
2001–2005	3 (1.1%)
2006–2010	24 (9.5%)
2011–2015	80 (31.9%)
2016–2021	171 (68.3%)
Previous treatments	
None	7 (2.5%)
Surgery	233 (82.9%)
Radiation therapy	168 (59.8%)
Chemotherapy	227 (80.8%)
Hormone therapy	138 (49.1%)
Immunotherapy	39 (13.9%)
Other	41 (14.6%)
Ongoing treatments	
None	161 (57.3%)
Surgery	0 (0.0%)
Radiation therapy	7 (2.5%)
Chemotherapy	14 (5.0%)
Hormone therapy	84 (29.9%)
Immunotherapy	13 (4.6%)
Other	19 (6.8%)
Use of mental health services (Yes)	72 (25.6%)

*SD* standard deviations

### Description of the Portuguese FACT-Cog-v3

Means, SDs, and range for the Portuguese version of the FACT-Cog-v3 items and subscales are presented in Table 2. The lowest score emerged for CogPCH2 (M = 1.21, SD = 1.13) and the highest score for CogM9 (M = 3.54, SD = 0.86). The mean scores of the four subscales were 47.56 (SD = 20.47), 13.64 (SD = 3.54), 16.33 (SD = 7.70), and 8.64 (SD = 4.51) for CogPCI, CogOth, CogPCA, and CogQOL, respectively.

**Table 2:** FACT-Cog-v3 subscales means and standard deviations, range, item-total correlations, and Cronbach’s alphas (N = 281)

	Mean (SD)	Range	Corrected item-total correlation	Cronbach’s alpha if item deleted	Cronbach’s alpha
FACT-Cog-v3					
Perceived Cognitive Impairments (CogPCI)	47.56 (20.47)	0–80			0.97
CogA1—I have had trouble forming thoughts	2.38 (1.37)	0–4	0.790	0.97	
CogA3—My thinking has been slow	2.22 (1.34)	0–4	0.803	0.97	
CogC7—I have had trouble concentrating	1.84 (1.30)	0–4	0.796	0.97	
CogM9—I have had trouble finding my way to a familiar place	3.54 (0.86)	0–4	0.464	0.97	
CogM10—I have had trouble remembering where I put things, like my keys or my wallet	2.28 (1.26)	0–4	0.716	0.97	
CogM12—I have had trouble remembering new information, like phone numbers or simple instructions	2.26 (1.38)	0–4	0.774	0.97	
CogV13—I have had trouble recalling the name of an object while talking to someone	2.41 (1.30)	0–4	0.779	0.97	
CogV15—I have had trouble finding the right word(s) to express myself	2.21 (1.36)	0–4	0.838	0.97	
CogV16—I have used the wrong word when I referred to an object	3.07 (1.18)	0–4	0.704	0.97	
CogV17b—I have had trouble saying what I mean in conversations with others	2.69 (1.28)	0–4	0.794	0.97	
CogF19——I have walked into a room and forgotten what I meant to get or do there	2.27 (1.18)	0–4	0.733	0.97	
CogF23—I have had to work really hard to pay attention or I would make a mistake	2.36 (1.35)	0–4	0.839	0.97	
CogF24—I have forgotten names of people soon after being introduced	2.54 (1.29)	0–4	0.624	0.97	
CogF25—My reactions in everyday situations have been slow	2.57 (1.21)	0–4	0.799	0.97	
CogC31—I have had to work harder than usual to keep track of what I was doing	2.09 (1.35)	0–4	0.828	0.97	
CogC32—My thinking has been slower than usual	2.24 (1.33)	0–4	0.804	0.97	
CogC33a—I have had to work harder than usual to express myself clearly	2.39 (1.34)	0–4	0.867	0.97	
CogC33c—I have had to use written lists more often than usual so I would not forget things	1.94 (1.35)	0–4	0.723	0.97	
CogMT1—I have trouble keeping track of what I am doing if I am interrupted	2.01 (1.36)	0–4	0.827	0.97	
CogMT2—I have trouble shifting back and forth between different activities that require thinking	2.26 (1.34)	0–4	0.801	0.97	
Comments from Others (CogOth)	13.64 (3.54)	0–16			0.92
CogO1—Other people have told me I seemed to have trouble remembering information	3.15 (1.12)	0–4	0.765	0.92	
CogO2—Other people have told me I seemed to have trouble speaking clearly	3.50 (0.97)	0–4	0.839	0.89	
CogO3—Other people have told me I seemed to have trouble thinking clearly	3.50 (0.94)	0–4	0.860	0.88	
CogO4—Other people have told me I seemed confused	3.49 (0.90)	0–4	0.831	0.90	
Perceived Cognitive Abilities (CogPCA)	16.33 (7.70)	0–36			0.93
CogPC1—I have been able to concentrate	1.99 (0.93)	0–4	0.678	0.93	
CogPV1——I have been able to bring to mind words that I wanted to use while talking to someone	2.25 (0.97)	0–4	0.736	0.93	
CogPM1—I have been able to remember things, like where I left my keys or wallet	2.06 (1.03)	0–4	0.718	0.93	
CogPM2—I have been able to remember to do things, like take medicine or buy something I needed	2.27 (1.00)	0–4	0.674	0.93	
CogPF1—I am able to pay attention and keep track of what I am doing without extra effort	1.89 (1.12)	0–4	0.809	0.92	
CogPCH1———My mind is as sharp as it has always been	1.40 (1.19)	0–4	0.792	0.92	
CogPCH2—My memory is as good as it has always been	1.21 (1.13)	0–4	0.738	0.93	
CogPMT1—I am able to shift back and forth between two activities that require thinking	1.69 (1.08)	0–4	0.817	0.92	
CogPMT2—I am able to keep track of what I am doing, even if I am interrupted	1.58 (1.08)	0–4	0.799	0.92	
Impact on QoL (CogQoL)	8.64 (4.51)	0–16			0.92
CogQ35—I have been upset about these problems	1.98 (1.25)	0–4	0.754	0.92	
CogQ37—These problems have interfered with my ability to work	2.25 (1.26)	0–4	0.845	0.89	
CogQ38—These problems have interfered with my ability to do things I enjoy	2.30 (1.24)	0–4	0.851	0.89	
CogQ41—These problems have interfered with the quality of my life	2.12 (1.26)	0–4	0.835	0.90	

*SD* standard deviations

### Factor validity

Mardia’s Test showed that data is not multivariate normal, g1p = 250.14, χSkew = 11,714.97, *p* < 0.001; g2p = 1351.46, ZKurtosis = 34.26, *p* < 0.001; χSMSkew = 11,847.45, *p* < 0.001. The achieved sample size was enough to ensure stability of a factor solution.

**Fig. 2:**
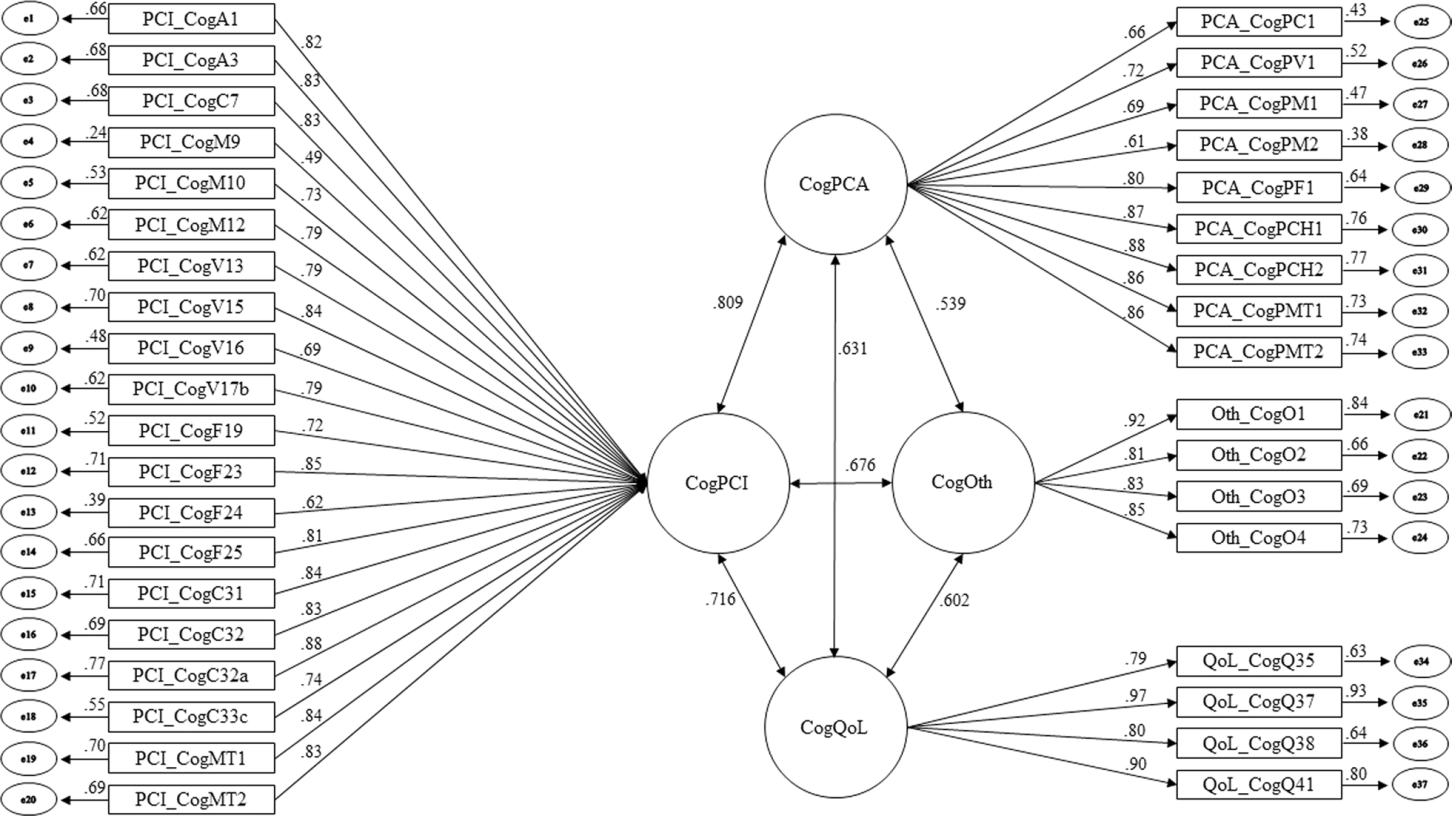
Diagram of four-factor structure (37 items) obtained using CFA with WLSMV estimator

A CFA with WLSMV was used to confirm the four-factor structure of the scale. Results revealed a good global adjustment, χ2(623) = 1096.48; CFI = 0.903; TLI = 0.897; RMSEA = 0.052, RMSEA 90% CI[0.047, 0.057]; SRMR = 0.055. Moreover, all items reached high factor weights and appropriate individual reliabilities on latent variables. The structural model that was tested using CFA and the resultant factor loadings and correlations are displayed in Fig. 2. Factor analysis of the 33-item FACT-Cog-v3 revealed a similar pattern (see Additional file 1).

### Reliability

For FACT-Cog-v3 dimensions of CogPCI, CogOth, CogPCA, and CogQoL, adequate mean inter-item correlations were obtained, 0.604, 0.755, 0.608, and 0.749, respectively, indicating that items in the same factor must be assessing the same construct. Regarding corrected item-total correlations, for each dimension, all items showed adequate item-total correlations (ranging between 0.464 and 0.867), indicating that all items are well correlated with the corresponding dimension. Cronbach’s alpha coefficient for FACT-Cog-v3 subscales were 0.97 for CogPCI, 0.92 for CogOth, 0.93 for CogPCA, and 0.92 for CogQoL, indicating high reliability. None of the items would substantially affect reliability if they were deleted, since all values are around the Cronbach’s alpha for each subscale. Internal consistency estimates for the FACT-Cog-v3 subscales are displayed in Table 2.

### Concurrent validity

Spearman’s correlations were calculated between the FACT-Cog-v3 subscales scores and the QLQ-C30 cognitive functioning subscale to establish concurrent validity (Table 3). All FACT-Cog-v3 subscales scores correlated positively (moderate and strong correlations) with QLQ-C30 cognitive functioning subscale.

**Table 3:** FACT-Cog-v3 Spearman’s correlations with cognitive functioning, anxiety, depression, fatigue, sleep disturbance, global health status, pain, and nausea/vomiting scores

	Cognitive functioning (QLQ-C30 cognitive functioning subscale)	Anxiety (HADS-A)	Depression (HADS-D)	Fatigue (QLQ-C30 fatigue subscale)	Sleep disturbance (QLQ-C30 sleep disturbance subscale)	Global health status (QLQ-C30 global health status subscale)	Pain (QLQ-C30 pain subscale)	Nausea/Vomiting (QLQ-C30 nausea/vomiting subscale)
FACT-Cog-v3								
Perceived Cognitive Impairments (CogPCI)	0.763 ***	− 0.516 ***	− 0.651 ***	− 0.463 ***	− 0.393 ***	0.401 ***	− 0.390 ***	− 0.222 ***
Comments from Others (CogOth)	0.528 ***	− 0.431 ***	− 0.464 ***	− 0.347 ***	− 0.328 ***	0.311 ***	− 0.283 ***	− 0.192 **
Perceived Cognitive Abilities (CogPCA)	0.698 ***	− 0.515 ***	− 0.635 ***	− 0.411 ***	− 0.364 ***	0.446 ***	− 0.346 ***	− 0.182 **
Impact on QoL (CogQoL)	0.650 ***	− 0.538 ***	− 0.660 ***	− 0.572 ***	− 0.500 ***	0.569 ***	− 0.479 ***	− 0.269 ***
Perceived Cognitive Impairments (CogPCI)§	0.482 ***	− 0.206 ***	− 0.313 ***	− 0.246 ***	− 0.185 **	0.088	− 0.203 ***	− 0.130 *
Perceived Cognitive Abilities (CogPCA)§§	0.240 ***	− 0.202 **	− 0.256 ***	− 0.085	− 0.095	0.231 ***	− 0.067	− 0.011

*FACT-Cog-v3* Functional Assessment of Cancer Therapy-Cognitive Function-Version 3; *QLQ-C30* European Organization for Research and Treatment of Cancer Quality of Life Questionnaire-Version 3 (EORTC or simply QLQ-C30); *HADS* Hospital Anxiety and Depression Scale. §Partial correlation controlling for CogPCA; §§Partial correlation controlling for CogPCI. ***p < 0.001; **p < 0.01; *p < 0.05

### Convergent and discriminant validity assessed by the Fornnel and Larcker method

According to the Fornell and Larcker [41] testing system, indicators of convergent validity showed good results: CR of the factors revealed adequate results, with 0.98, 0.95, 0.96, and 0.96 for CogPCI, CogOth, CogPCA, and CogQoL, respectively; and AVE showed adequate values (AVE_CogPCI_ = 0.63, AVE_CogOth_ = 0.74, AVE_CogPCA_ = 0.62, AVE_CogQoL_ = 0.75).

Discriminant validity was assessed by comparison of AVEs with the square of the correlation between factors. All factors have discriminant validity, as AVE values were above the square of the correlation between the factors, except between CogPCI and CogPCA (Table 4). Further analyses were conducted to examine if the two factors should be maintained as separate dimensions. As we can see in Table 3, when we conduct partial correlations between CogPCI and the QLQ-C30 global health status controlling for CogPCA, results are not significant; contrarily, between CogPCA and the same QLQ-C30 subscale controlling for CogPCI, the results are significant.

**Table 4:** Discriminant validity results—Fornell and Larcker criterion

	CogPCI	CogOth	CogPCA	CogQoL
Perceived Cognitive Impairments (CogPCI)	**0.63**			
Comments from Others (CogOth)	0.46	**0.74**		
Perceived Cognitive Abilities (CogPCA)	0.65	0.29	**0.62**	
Impact on QoL (CogQoL)	0.51	0.36	0.40	**0.75**

Bold numbers on the diagonal are AVEs and off diagonal numbers are squared interconstruct correlations

### Convergent and discriminant validity by external criteria

Convergent validity was examined through Spearman’s correlation between FACT-Cog-v3 subscales and HADS and QLQ-C30 fatigue, sleep disturbance, and global health status subscales (Table 3). All FACT-Cog-v3 scores correlated negatively (moderate correlations) with HADS-A and HADS-D and with QLQ-C30 fatigue and sleep disturbance subscales and correlated positively (moderate correlations) with QLQ-C30 global health status subscale.

For discriminant validity, correlations between all FACT-Cog-v3 scores and QLQ-C30 pain and nausea/vomiting subscales were obtained, showing negative correlations (weak and moderate for pain and weak for nausea/vomiting) (Table 3).

## Discussion

The main goal of the present study was to provide evidence of the reliability and validity of the Portuguese version of the FACT-Cog-v3, thus making available an instrument that assesses PCF to the Portuguese cancer population. Our results demonstrated that the FACT-Cog-v3 is a reliable and valid measure of CRCI among patients with non-CNS cancers in Portugal.

In line with recent recommendations arising from the positive results of Koch et al. [19] study and FACIT scoring instructions, this study used the full 37-item scale, including the additional multitasking items. The findings of the CFA showed a good fit between the hypothesized model and the observed data, as well as acceptable loadings. Thus, all items measuring the factors support the four-factor structure for the FACT-Cog-v3 scale, consistent with the CogPCI, CogQoL, CogOth, and CogPCA subscales proposed by the original authors [7] and other language validations [16, 17]. The results also confirmed that the additional multitasking items load with the expected subscales, as proposed by the original authors [7]. Considering the positive results obtained with the 37 items, this study supports the use of the full scale in research and clinical practice to gain a comprehensive understanding of PCF [19]. We should also note that good psychometric findings were obtained with the 33-item version, and conclude that both Portuguese versions are valid. Thus, each user can opt for the version that best fits their purpose. Moreover, this validation study was conducted with patients with non-CNS cancers, rather than with breast cancer patients only, as most studies previously did [15–17], providing support to the robustness and stability of the instrument’s multidimensional structure, which is transversal across various cultural contexts and cancer populations.

Furthermore, there was evidence for convergent and discriminant validity of the four-factor model: the results showed a positive correlation between the items of each of the factors and showed that the items from each subscale did not correlate with items of the other subscales, respectively. We should note, however, that although the findings point towards good discriminant validity between factors, there is an exception for CogPCI and CogPCA, with values slightly above the desired. Nonetheless, the literature affirms that these scales represent two separate factors [43] and the results obtained in the present work for partial correlations show that both factors are important to measure different information related to QoL. Therefore, we decided to maintain both factors as separate dimensions, in line with the original scale.

Reliability results supported the dimensionality findings. Our findings indicated very good internal consistency for the factors of the FACT-Cog-v3 (all above 0.91), in line with or even higher than reliability scores found in previous studies [16]. At the item level, all items appeared to be worthy of retention, and the inter-item and item-total correlations indicated the items’ adequacy and homogeneity in measuring the construct that the FACT-Cog-v3 intends to. The values of the Cronbach’s alpha coefficients also did not improve with the removal of any of the items on the four factors. Taken together, these results confirm the theoretical structure with the four subscales.

Results obtained from concurrent validity analysis revealed that all FACT-Cog-v3 subscales scores had moderate and strong positive correlations with the QLQ-C30 cognitive functioning subscale. The QLQ-C30 cognitive functioning subscale is an established self-report scale to demonstrate concurrent validity of the FACT-Cog-v3 [16, 19]. This result is thus consistent with the moderate correlations found between the Chinese [15] and Korean [16] versions of the FACT-Cog-v3 and the QLQ-C30 cognitive functioning subscale, providing support for the concurrent validity of the Portuguese version of the FACT-Cog-v3.

Similar to the other validations of the FACT-Cog-v3, evidence of convergent validity of the scale was confirmed by correlations of this scale with theoretically related constructs. Moderate negative correlations were found with anxiety [3, 15, 44] and depressive [3, 16, 44] symptoms, fatigue [3, 15, 19, 44], and sleep disturbance [3, 44]. Moderate positive correlations were found for global health status [15]. These findings are consistent with previous validation studies [15, 16, 19]. In terms of discriminant validity, weak and moderate negative correlations were obtained for pain and weak negative correlations for nausea/vomiting, as described in Koch et al. [19]. Thus, these results provide further evidence of the FACT-Cog-v3’s discriminant validity.

Despite the encouraging results, this study has some limitations that should be addressed. First, our sample was recruited online, which may represent a selection bias (i.e., selection of those cancer patients who have digital literacy, access to the Internet, and perhaps are more educated and employed). Therefore, future research should recruit participants in-person, to examine if the good psychometric properties verified in this study are maintained with cancer patients with different sociodemographic characteristics. The study’s cross-sectional design is also a limitation, constraining the determination of test–retest reliability. We recommend that the temporal stability of this version should also be examined in the future. In addition to temporal stability, measurement invariance across groups (e.g., sex, age), namely metric, configural, and scalar, should be performed in future studies. Additionally, future studies should consider performing these analyses with bigger samples. Finally, caution is also needed in interpreting these findings, considering the social and health context of the COVID-19 Pandemic in which the study was conducted, since some authors alert for the possible interference of the stress related to this event on cognitive problems reported by cancer survivors [45] and the impact of the COVID-19 disease on cognitive functioning [46]. However, a previous preliminary study conducted outside the context of Pandemic [25] point to similar results, which leads us to believe that it may not have an influence on the validation of the scale.

Notwithstanding these limitations, we believe that our study provides important contributions to the field of CRCI literature, offering evidence of the good psychometric characteristics of the FACT-Cog-v3 scale in a Portuguese sample of patients with non-CNS cancers. Using this measure in clinical practice may contribute to a better understanding of patients’ cognitive difficulties, thus helping to provide proper interventions to mitigate the effects of CRCI and improve QoL in this population. Furthermore, future studies can also use the Portuguese version of the FACT-Cog-v3 to assess the efficacy of cognitive intervention programs in cancer patients.

## Conclusions

Cognitive symptoms are one of the most frequent and worrying side effects experienced by patients with non-CNS cancers. Considering its detrimental impact on QoL, it is necessary to provide validated instruments to help researchers and clinicians evaluate the nature and extent of these complaints. This study aimed to analyze the psychometric properties of the Portuguese version of the FACT-Cog-v3. Overall, the 37-items four-factor structure of the scale appears to be a reliable and valid measure of CRCI among patients with non-CNS cancers in Portugal.

## Supplementary Information

**Additional file 1**. Factor analysis of the 33-item FACT-Cog-v3.

## Data Availability

The datasets generated during and/or analyzed during the current study are available from the corresponding author on reasonable request.

## Abbreviations

AVE: Average variance extracted
CRCI: Cancer-related cognitive impairment
CNS: Central nervous system
CogPCA: Perceived cognitive abilities
CogPCI: Perceived cognitive impairments
CogOth: Comments from others
CogQoL: Impact on quality of life
CFI: Comparative Fit Index
CFA: Confirmatory factor analysis
CR: Construct reliability
EORTC QLQ-C30-version 3/QLQ-C30: European Organization for Research and Treatment of Cancer Quality of Life Questionnaire Core-30
FACIT: Functional Assessment of Chronic Illness Therapy
FACT-Cog-v3: Functional Assessment of Cancer Therapy-Cognitive Function-Version 3
HADS: Hospital Anxiety and Depression Scale
PCF: Perceived cognitive functioning
R^2^: Individual reliability
RMSEA: Root Mean Square Error of Approximation
SD: Standard deviations
SRMR: Standardized Root Mean Square Residual
TLI: Tucker–Lewis Index
QoL: Quality of life
WLSMV: Weighted least squares with mean and variance adjustment
*χ*^2^: Chi-square
