# Supplementary material for: Validation study of the Functional Assessment of Cancer Therapy-Cognitive Function-Version 3 for the Portuguese population
Source: BMC Psychol. 2022 Dec 14;10:305. doi: 10.1186/s40359-022-01018-w (PMC9748889; doi:10.1186/s40359-022-01018-w)
Supplement: Supplementary file 1 — Additional file 1. Factor analysis of the 33-item FACT-Cog-v3. [file 40359_2022_1018_MOESM1_ESM.docx]

**Additional File 1 – Factor analysis of the 33-item FACT-Cog-v3**

**Summary of Model Fit Statistics for Confirmatory Factor Analysis (CFA) for the 33-item FACT-Cog-v3**

| χ2 | df | CFI | TLI | RMSEA | RMSEA 90% CI | SRMR |
| --- | --- | --- | --- | --- | --- | --- |
| 919.920 | 489 | 0.896 | 0.888 | 0.056 | [0.051, 0.062] | 0.056 |

χ2=Chi-Square; CFI=Comparative Fit Index; TLI=Tucker-Lewis Index; RMSEA=Root Mean Square Error of Approximation; SRMR=Standardized Root Mean Square Residual.

**
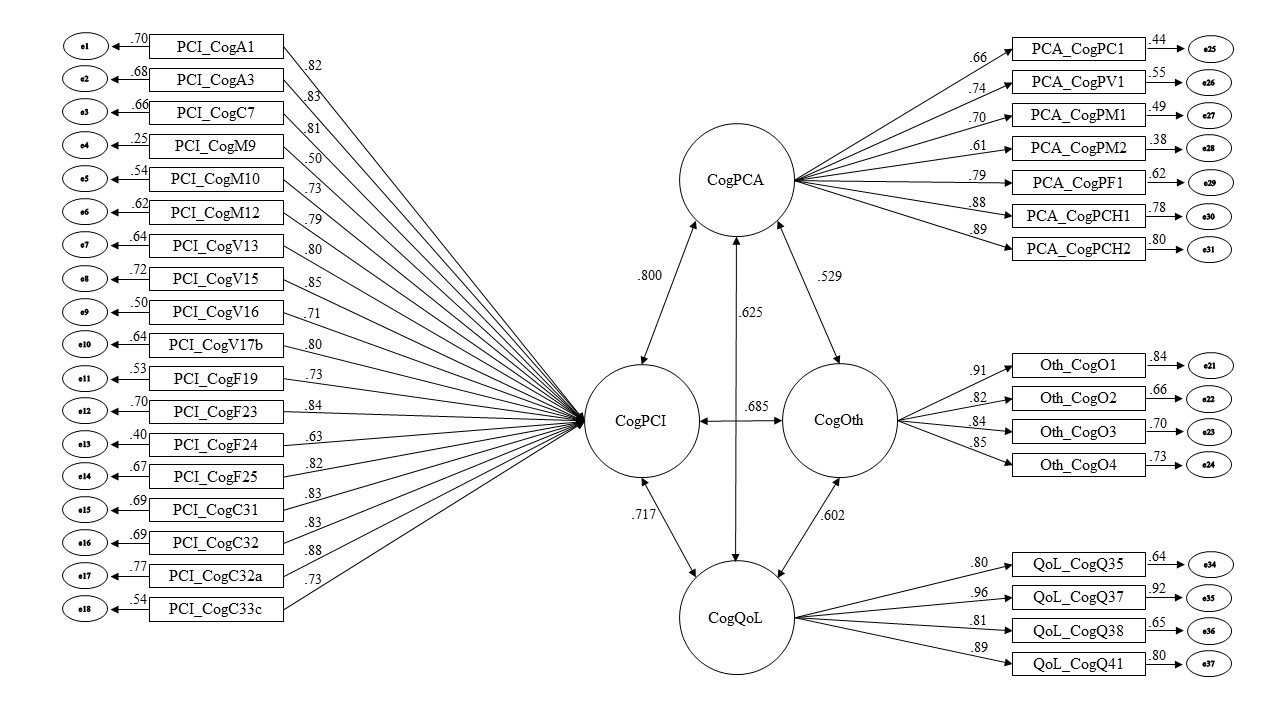
Diagram of four-factor structure obtained using CFA with WLSMV estimator for the 33-item FACT-Cog-v3**
